# Angiotensin-Converting Enzyme Inhibitor-Induced Angioedema Following Posterior Reversible Encephalopathy Syndrome in a Child: A Case Report

**DOI:** 10.7759/cureus.31568

**Published:** 2022-11-16

**Authors:** Kanami Ichikawa, Daisuke Matsuoka, Tsubasa Murase, Yozo Nakazawa

**Affiliations:** 1 Department of Pediatrics, Nagano Red Cross Hospital, Nagano, JPN; 2 Department of Pediatrics, Shinshu University School of Medicine, Matsumoto, JPN

**Keywords:** pediatrics, posterior reversible encephalopathy syndrome (pres), dry cough, angiotensin-converting enzyme inhibitor, angioedema

## Abstract

Angioedema is a rare but potentially lethal side effect of angiotensin-converting enzyme inhibitors (ACEIs). Most ACEI-induced angioedema (ACEI-AE) cases have been reported in adults, with few reports of cases in children. Posterior reversible encephalopathy syndrome (PRES) is a neurological disorder that presents with acute onset of symptoms, including headache, vomiting, visual disturbances, and seizures. We report the case of a patient who developed ACEI-AE after developing PRES during the treatment of steroid-resistant nephrotic syndrome. ACEI-AE is very rare, especially in children, but can be life-threatening if swelling of the tongue or the throat blocks the airway. Whenever ACEIs are used, even in children, clinicians should be aware of the possibility of the occurrence of ACEI-AE, particularly when accompanied by dry cough. Moreover, bradykinin may be associated with PRES onset in patients with ACEI-AE and may be a risk factor for PRES.

## Introduction

Angiotensin-converting enzyme inhibitors (ACEIs) are widely used to treat cardiovascular and renal disorders in both adults and children. Angioedema is a rare but potentially lethal side effect of ACEIs, with an incidence rate of 0.1-0.7% [1]. ACEI-induced angioedema (ACEI-AE) is characterized by non-pitting edema of the skin and/or mucous membranes, most commonly in the head and neck area, often involving swelling of the lips and/or tongue [1]. ACEI-AE has mainly been reported in adults, with few reports in children [2-6].

Posterior reversible encephalopathy syndrome (PRES) is a neurological disorder characterized by varied neurological symptoms, including headache, vomiting, visual disturbances, and seizures [7,8]. Brain imaging usually reveals vasogenic edema predominantly involving the bilateral parieto-occipital regions [7]. The pathophysiology of PRES is not clearly understood; however, cerebral vasogenic edema due to blood-brain barrier dysfunction and endothelial dysfunction are thought to be the main causes [8]. To our knowledge, there are no previous reports on the association between ACEI-AE and PRES.

Here, we report the case of a patient with steroid-resistant nephrotic syndrome who developed ACEI-AE following PRES. Written informed assent for the publication of clinical details was obtained from the parents.

## Case presentation

A three-year-old Japanese girl was referred to our hospital with steroid-resistant nephrotic syndrome. Percutaneous renal biopsy showed minor glomerular abnormalities; light microscopy findings showed some enlarged epithelial and peduncular cells of Bowman’s sac in the glomerulus at the dermo-medullary border. Electron microscopy findings showed flattening of the foot process. Cyclosporine A and prednisolone were administered. Proteinuria gradually decreased, and edema resolved after one week.

**Figure 1:**
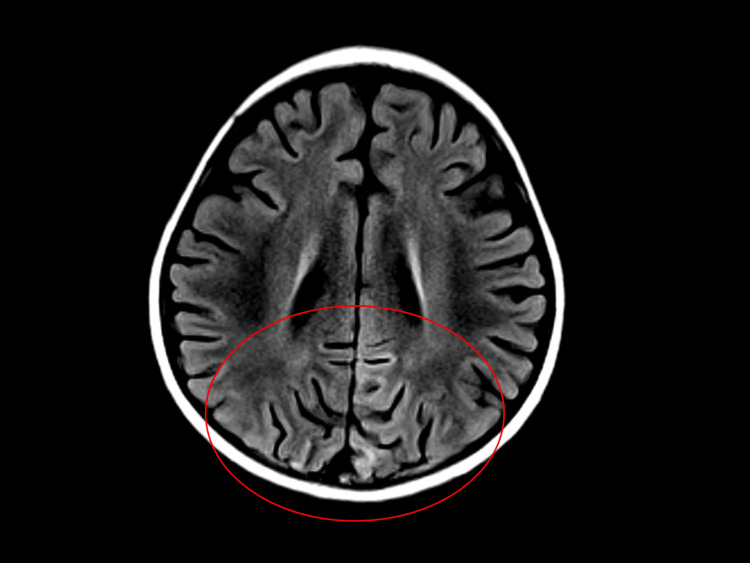
Brain FLAIR magnetic resonance imaging Hypertense signals in the cortical and subcortical white matter of bilateral occipital lobes (red circle) in a FLAIR sequence. FLAIR, fluid-attenuated inversion recovery

At two weeks after cyclosporine A administration, her blood pressure gradually rose to 114/76 mmHg (Stage 1; the 95th percentile was 106/64 mmHg; Stage 2; 95th percentile + 12 mmHg was 118/76 mmHg [9]). The dose of cyclosporine A was reduced from 100 mg to 80 mg (5 mg/kg) per day because the cyclosporine A trough level was 233 ng/mL. Enalapril administration (0.07 mg/kg/day) was initiated instead of calcium channel blocker administration owing to its potential to increase the blood levels of cyclosporine. After two days, she developed a dry cough from enalapril. Her medical history was unremarkable except for febrile convulsions. The patient’s family history was unremarkable. She had previously developed a skin rash after exposure to amoxicillin. Two days after the dry cough onset, she developed headaches, vomiting, and seizures. Her blood pressures before and during seizures were 106/70 mmHg and 142/111 mmHg, respectively, and edema was absent. Seizures stopped after midazolam administration, and blood pressure was controlled with nicardipine. Magnetic resonance imaging revealed hyperintense signals in the cortical and subcortical white matter of bilateral occipital lobes in a fluid-attenuated inversion recovery sequence (Figure 1).

Kidney function and serum electrolyte levels were within normal limits. The serum albumin level was 1.8 g/dL (1.0 g/dL 4 days prior), and the cyclosporine A trough level was 199 ng/mL (Table 1). PRES was diagnosed. The following day, the patient regained consciousness, and her blood pressure remained normal without nicardipine.

**Table 1:** Laboratory results PRES, posterior reversible encephalopathy syndrome

	4 days before the onset of PRES	At the onset of PRES	Reference range
Total protein	3.3	4.9	6.0–7.7 g/dL
Albumin	1.0	2.0	4.1–5.1 g/dL
Blood urea nitrogen	25.2	12.1	5.5–19.3 mg/dL
Creatinine	0.20	0.18	0.20–0.40 mg/dL
Sodium	134	136	136–144 mmol/L
Potassium	4.7	3.9	3.6–4.8 mmol/L
Chloride	104	101	101–110 mmol/L
C-reactive protein	0.00	0.09	0.00–0.14 mg/dL
Cyclosporine A (trough)	233	199	Variable, ng/mL
Urine Protein/Creatinine ratio	5.13	0.86	<0.15 g/g Creatinine

**Figure 2:**
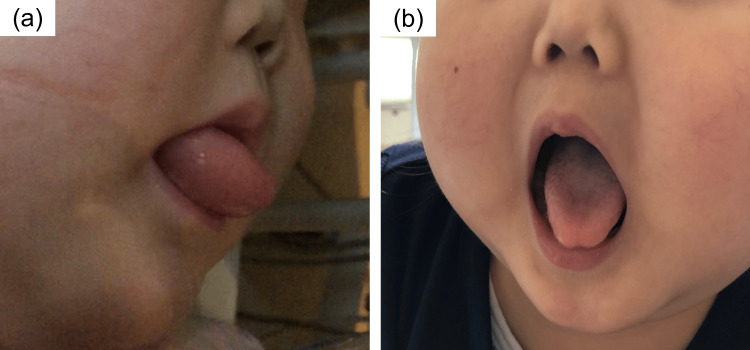
Angioedema due to enalapril (a) Edema with protrusion of the tongue. (b) Complete resolution of angioedema

Two days later (the 7th day after initiating enalapril), the patient developed swelling of the lips and tongue. The tongue protruded from her mouth (Figure 2). Her vital signs were as follows: temperature, 37.7°C; pulse rate, 148 beats/min; respiratory rate, 16 breaths/min; blood pressure, 108/58 mmHg; and pulse oximetry, 94% on 2 L oxygen via nasal cannula. Snoring was noted; however, stridor and wheezing were not audible, and no cutaneous lesions were present. The swelling resolved on its own within several hours but recurred the following day. Angioedema due to enalapril was suspected, leading to enalapril discontinuation. The serum complement C3, C4, and C1 esterase inhibitor levels were within the normal limits (Table 2). She developed no further episodes of angioedema.

**Table 2:** Complement test results

	Value	Reference range
Complement C3	104	73–138 mg/dL
Complement C4	15.1	11–31 mg/dL
C1 esterase inhibitor	111	<130%

## Discussion

ACEI-AE is postulated to be mediated by bradykinin and/or substance P accumulation due to angiotensin-converting enzyme (ACE) inhibition [1]. As bradykinin is degraded by ACE, inhibition of ACE with ACEI drugs leads to increased bradykinin levels. Bradykinin causes vasodilation and increases vascular permeability. This results in fluid extravasation into the tissues and edema development, thus leading to angioedema [1]. The patient developed ACEI-induced dry cough before the onset of angioedema. ACEI-induced dry cough is postulated to be caused by increased levels of bradykinin, substance P, and other factors [10] and has been reported to be an independent risk factor for ACEI-AE [11]. Patients who receive ACEI drugs, particularly those who develop ACEI-induced dry cough, should be monitored carefully because they are likely to develop ACEI-AE. In addition, ACEI-AE often occurs within the first week of the initiation of ACEIs but can also occur months to years later [12]. ACEI-AE can be life-threatening if swelling occurs around the tongue or throat. Clinicians should provide proper education to patients and/or families when using ACEIs.

Our patient developed PRES prior to the onset of ACEI-AE. The mechanism of PRES has not yet been elucidated, but cerebral vasogenic edema is thought to be the main cause owing to blood-brain barrier dysfunction and endothelial dysfunction [8]. Blood-brain barrier dysfunction may be caused by hypertension. Especially, hypertension above the upper limit of cerebral blood flow autoregulation leads to cerebral hyperperfusion and blood brain barrier dysfunction [8]. Endothelial dysfunction is generally caused by circulating endogenic factors (such as in preeclampsia and sepsis) and exogenic factors (such as calcineurin inhibitors or cytotoxic medications), leading to increased vascular permeability and vasogenic edema [8]. In our case, the patient's cyclosporine A trough level was 199 ng/mL at the onset of PRES; therefore, cyclosporine A might have contributed to the development of PRES. However, the patient’s blood pressure was normal (106/70 mmHg) prior to PRES onset. Although her blood pressure was elevated during seizures (142/111 mmHg), this increase was considered temporary as the patient did not need nicardipine the next day. Therefore, it is not known how much this temporary increase in blood pressure contributed to the development of PRES.

Given that the development of PRES is attributed to increased vascular permeability, bradykinin may also be involved. In our case, the patient had a dry cough caused by ACEI-AE when she developed PRES. Thus, it is postulated that there was an increase in vascular permeability due to bradykinin. In terms of ACEI-AE, the bradykinin levels have been shown to increase more than 10-fold in patients with ACEI-AE compared with that in healthy controls [13]. Therefore, ACEI-AE can be responsible for the development of PRES. This case indicates that increasing vascular permeability by bradykinin may be important for the development of PRES. However, as no studies have directly documented the relationship between ACEI-AE and PRES, as well as the relationship between ACEI and PRES, the possibility that it happened by chance cannot be ruled out. Further studies are needed to clarify the association between ACEI-AE and PRES.

## Conclusions

ACEI-AE is very rare, especially in children, but can be life-threatening if swelling of the tongue or throat blocks the airway. Whenever ACEIs are used, clinicians should be aware of the potential for the occurrence of ACEI-AE, even in children. ACEI-AE and ACEI-induced dry cough are postulated to be caused by increased levels of bradykinin, and ACEI-induced dry cough is reported to be an independent risk factor for ACEI-AE. Patients who develop ACEI-induced dry cough should be monitored carefully. Moreover, considering that bradykinin is postulated to increase vascular permeability, bradykinin may be associated with PRES onset in patients with ACEI-AE and may be a risk factor for PRES.
